# Ethnoracial disparities in childhood growth trajectories in Brazil: a longitudinal nationwide study of four million children

**DOI:** 10.1186/s12887-024-04550-3

**Published:** 2024-02-10

**Authors:** Helena Benes Matos da Silva, Rita de Cássia Ribeiro-Silva, Juliana Freitas de Mello e Silva, Irina Chis Ster, Poliana Rebouças, Emanuelle Goes, Maria Yury Ichihara, Andrêa Ferreira, Julia M. Pescarini, Rosemeire Leovigildo Fiaccone, Enny S. Paixão, Maurício L. Barreto

**Affiliations:** 1 School of Nutrition, Federal University of Bahia, Salvador, BA Brazil; 2 Center for Data and Knowledge Integration for Health (CIDACS), Gonçalo Moniz Institute, Oswaldo Cruz Foundation, Edf. Tecnocentro, Sl. 315. Rua Mundo, 121. Trobogy, Salvador, BA 41745-715 Brazil; 3 Infection and Immunity Research Institute, St George’s University of London, London, UK; 4 The Ubuntu Center on Racism, Global Movements, and Population Health Equity, Dornsife School of Public Health, Drexel University, Philadelphia, USA; 5 Epidemiology and Population Health, London School of Hygiene and Tropical Medicine, London, UK; 6 Department of Statistics, Federal University of Bahia, Salvador, BA Brazil

**Keywords:** Ethnic-racial groups, Child growth trajectories, Food and nutrition surveillance system, Racism, Race

## Abstract

**Background:**

The literature contains scarce data on inequalities in growth trajectories among children born to mothers of diverse ethnoracial background in the first 5 years of life.

**Objective:**

We aimed to investigate child growth according to maternal ethnoracial group using a nationwide Brazilian database.

**Methods:**

A population-based retrospective cohort study employed linked data from the CIDACS Birth Cohort and the Brazilian Food and Nutrition Surveillance System (SISVAN). Children born at term, aged 5 years or younger who presented two or more measurements of length/height (cm) and weight (kg) were followed up between 2008 and 2017. Prevalence of stunting, underweight, wasting, and thinness were estimated. Nonlinear mixed effect models were used to estimate childhood growth trajectories, among different maternal ethnoracial groups (White, Asian descent, Black, Pardo, and Indigenous), using the raw measures of weight (kg) and height (cm) and the length/height-for-age (L/HAZ) and weight-for-age z-scores (WAZ). The analyses were also adjusted for mother’s age, educational level, and marital status.

**Results:**

A total of 4,090,271 children were included in the study. Children of Indigenous mothers exhibited higher rates of stunting (26.74%) and underweight (5.90%). Wasting and thinness were more prevalent among children of Pardo, Asian, Black, and Indigenous mothers than those of White mothers. Regarding children’s weight (kg) and length/height (cm), those of Indigenous, Pardo, Black, and Asian descent mothers were on average shorter and weighted less than White ones. Regarding WAZ and L/HAZ growth trajectories, a sharp decline in average z-scores was evidenced in the first weeks of life, followed by a period of recovery. Over time, z-scores for most of the subgroups analyzed trended below zero. Children of mother in greater social vulnerability showed less favorable growth.

**Conclusion:**

We observed racial disparities in nutritional status and childhood growth trajectories, with children of Indigenous mothers presenting less favorable outcomes compared to their White counterparts. The strengthening of policies aimed at protecting Indigenous children should be urgently undertaken to address systematic ethnoracial health inequalities.

**Supplementary Information:**

The online version contains supplementary material available at 10.1186/s12887-024-04550-3.

## Background

Birth weight and infant growth are important markers of child health and future well-being [1–4]. Some conditions, such as premature birth, low birth weight and maternal malnutrition, have been well-documented factors associated with growth trajectory [5] while others, including socioeconomic status (SES), have been a consistent object of study [6–9].

In recent years a growing body of evidence has reported ethnoracial inequalities regarding infant growth and development [7, 10]. Race is a social construct that functions as an essential tool of racism, to separate and create social hierarchy, which has produced and reinforced segregation, differential quality and access to health care and unequal distributions of social determinants of health [11]. The ethnoracial inequities affecting mothers can also impact childhood outcomes [12]. Differences in rates of child survival among racial groups have been reported in Brazil [13, 14]. A study investigating mortality risk of children under 5 years of age by maternal self-declared race/ethnicity of over 19 million newborn babies in Brazil found that children born to Indigenous mothers had a 16-time higher risk of death due to malnutrition than their White counterparts [10]. Similarly, those born to Black or Pardo had over 2-times the risk of death due to malnutrition than their White counterparts [10].

Even though previous studies have evidenced the effects of racism and its manifestation on perinatal outcomes and child mortality, the literature on child growth outcomes by ethnoracial groups over time is scarce. Understanding the effects of ethnoracial inequities on growth trajectories requires thorough investigation to inform policy decision-making aimed at reducing inequalities and adequately achieving the 2025 global nutrition targets outlined by the World Health Organization (WHO) [11] and the United Nation’s 2030 Sustainable Development Goals (SDG) (eradication of hunger and all forms of malnutrition) [12]. The present study aims to investigate child growth according to maternal ethnoracial group using a nationwide Brazilian database.

## Methods

A population-based retrospective cohort study was conducted using data linked from two different Brazilian databases: (i) the CIDACS Birth Cohort [13], and (ii) the Food and Nutrition Surveillance System (SISVAN). The data consisted of children aged 0 to 60 months of age, born between January, 2003 to November, 2015, and followed up from January, 2008 until December, 2017. Details regarding the linkage process performed are available in previous publication [14].

The CIDACS Birth Cohort resulted from the linkage of the Live Birth Information System (SINASC) and the 100 Million Brazilian Cohort baseline. SINASC coverage extends to over 97% of live births in Brazil, with records collected through the Declaration of a Live Birth by a health professional present during the child’s delivery. This legally standardized form includes information about the parents, such as the mother’s name, age, local of residence, marital status, educational level. Also, pregnancy details, such as length of gestation, number of prenatal visits, type of delivery, and characteristics of the newborn, including sex, birth weight, congenital anomalies, and other factors [13].

The 100 Million Brazilian Cohort baseline was developed using administrative records from low-income individuals, whose families applied for the National Unified Register for Social Programs (*Cadastro Único*). This baseline variables encompasses a range of socioeconomic and demographic characteristics [15].

Since 2008, SISVAN has been monitoring the nutritional status of the Brazilian population by routinely recording individual-level sociodemographic, anthropometric (length/height and weight measurements), and food consumption data from users of public health services in all stages of life. This database includes data collected by primary health care professionals from individuals under care of the Brazilian Unified Health System (SUS), anthropometric data recorded of people benefiting from cash transfer program (Bolsa Família Program), and data from the e-SUS Primary Care strategy [16]. They use the equipment available at the unit, which can be a digital scale, a pediatric scale, an anthropometer, or a child anthropometer. The procedures for anthropometric measurement follows the protocols established by the Brazilian Ministry of Health [17]. The SISVAN data quality showed improvement over the period of 2008 to 2017 with completeness to almost 100% for height and weight along the years, and coverage ranging from 17.7 to 45.4% among SUS users [18].

The present study protocol was approved by the institutional review boards of the Collective Health Institute of the Federal University of Bahia (reference number 41695415.0.0000.5030) and the School of Nutrition, Federal University of Bahia (reference number 67205423.6.0000.5023).

### Study population

**Fig. 1:**
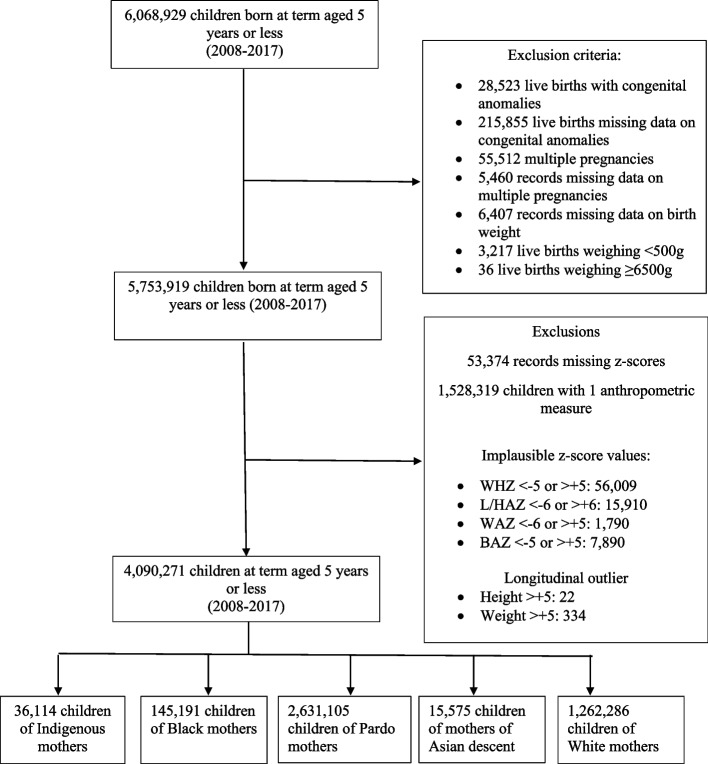
Flowchart detailing database construction and relevant exclusion criteria

We followed up singleton children born at term from birth up to the age of 60 months for whom two or more measurements of length/height (cm) and weight (kg) were recorded. Children with congenital anomalies or missing information on this characteristic, no recorded gestational age, and no birth weight were excluded. Implausible birth weights (< 500 g or ≥ 6500 g) were also excluded [19]. In an attempt to avoid bias in the analysis of low-birth-weight cases, multiple pregnancies were removed. We also excluded children with implausible z-scores for the following anthropometric variables: length/height-for-age z-scores (L/HAZ) < − 6 or > 6, weight-for-age z-scores (WAZ) < − 6 or > 5, weight-for-length/height z-scores (WHZ) < − 5 or > 5 and body-mass-index-for-age z-scores (BAZ) < − 5 or > 5, as these values are considered implausible under WHO recommendations [20]. Then, longitudinal outliers for height (<− 5/> + 5) and weight (<− 5/> + 5) were excluded [21] (Fig. 1).

### Exposure: race/ethnicity

Information on maternal race or skin color was obtained from the Brazilian Unified Registry for Social Programs at the CIDACS Birth Cohort. The adopted categories for race or skin color were those defined by the Brazilian Institute of Geography and Statistics (IBGE), which classifies racial groups as White, Black, Pardo, Yellow (which will be referred as Asian descent), and Indigenous [22]. For the purposes of the present analysis, “White” was adopted as a reference category, similarly to a previous study that indicated better overall health outcomes for children born to mothers who self-identified as White [23].

### Outcome: nutritional status and growth outcome

Information on the included children’s length/height (cm) and weight (kg) were retrieved from records in the SISVAN database.

Age (months) was calculated considering the time elapsed between date of birth and the date of the visit recorded in the SISVAN. Child sex (male or female) was obtained from SINASC registry.

The growth trajectories were estimated continuously as length/height (cm) and weight (kg), and with the standardized measures length/height-for-age z-scores (L/HAZ) and weight-for-age z-scores (WAZ), calculated according to the WHO Child Growth Standards [24]. Additionally, the nutritional status of the children was classified as stunting (low height-for-age), underweight (low weight-for-age), wasting (low weight-for-height), and thinness (low body-mass-index-for-age) using the WHO reference < − 2 SD z-score cutoff point [24].

### Maternal characterization

Maternal characteristics were available at the CIDACS Birth Cohort and the following covariates pertaining to mothers were described: maternal area of residence (rural or urban), household overcrowding (≤2 or > 2 people per room, calculated dividing the total number of people who live in the same house by the number of rooms), maternal education level (≤3 years, 4–7 years, ≥8 years of formal schooling), marital status (single, married/stable union, divorced/widowed), number of prenatal visits (none, 1–3, 4–6, ≥7 visits), type of delivery (cesarean or vaginal birth), and maternal age categorized for descriptive analyzes (10–13, 14–19, 20–34, 35–50 years) and continuous for modeling approaches.

### Statistical analysis

Initially, a descriptive analysis was performed considering maternal and child characteristics categorized according to ethnoracial groups [absolute value (n) and frequency (%) for categorical variables]. The prevalence of stunting, underweight, wasting, and thinness were calculated within each ethnoracial group. After removing missing observations non-linear mixed-effect (NLME) models were used to estimate length/height and weight trajectories in children by age since birth, with sex as a covariate [25]. Additional analyses were performed to investigate the trajectories of both L/HAZ and WAZ variables involving a mixed-effect model with cubic splines and eight knots (placed at age 2, 3, 6, 12, 18, 24, 36 and 58 months). The structure of NLME models includes both fixed and random effects; the former informs characteristics related to the population under study, while the latter accounts for the subject-specific associated variability of trajectories [25]. All models were adjusted for relevant confounding variables [maternal age (continuous variable), maternal education level and maternal marital status] [26, 27]. We also explored the possibility of interactions between child age vs race to examine to what extent growth trajectories differed by race/skin color [28].

The goodness of fit of the models was evaluated via training – with 70% of subjects - and testing – accounting for the remaining 30% - method. Sample procedure was performed in accordance with sex and maternal race/skin color. Measurement distributions for each sex were similar between both training and testing databases. All models were adjusted for maternal age, education level, and marital status.

Predictions from the most complex models representing the average fixed effects stratified by various explanatory variables and their uncertainties have been plotted. Analyzes were performed in R (for server version 4.1).

## Results

We included 4,090,271 children in this study; 64.33% were born to Pardo mothers, 30.86% to White mothers, 3.55% to Black mothers, 0.88% to Indigenous mothers, and 0.38% to Asian descent mothers. The characteristics of the study population by maternal ethnoracial group are reported in Table 1. Almost all of them resided in urban areas (with the notable exception of Indigenous women, of whom 72.83% lived in rural areas) and in less favorable housing conditions (30.04%). Indigenous (27.52%) and Black mothers (13.76%) had lower levels of formal schooling. Almost half of the Indigenous mothers were single or divorced/widowed (53.42%). An inadequate number of prenatal visits (< 7) were predominantly reported among Indigenous mothers (67.44%), followed by Pardo (48.55%), and Black (47.02%) mothers. While approximately 46.60% of live births to White mothers were delivered by cesarean section, this proportion was 17.48% for Indigenous women (Table 1).

**Table 1:** Distribution of maternal and child characteristics according to maternal race / skin color, 2008–2017

Variable	Categories	Children of White mothers (1262286)	%	Children of Asian descent mothers (15575)	%	Children of Black mothers (145191)	%	Children of Pardo mothers (2631105)	%	Children of Indigenous mothers (36114)	%	Total (4090271)
Birthweight, kg												
	< 2.5 kg	46,393	3.68	598	3.84	6729	4.63	95,589	3.63	1461	4.05	150,770
	≥ 2.5 kg	1,215,893	96.32	14,977	96.16	138,462	95.37	2,535,516	96.37	34,653	95.95	3,939,501
	Missing	–		–		–		–		–		–
Child’s gender												
	Male	616,678	48.85	7771	49.89	73.437	50.58	1,315,759	50.01	17,846	49.42	2,031,491
	Female	645,608	51.15	7804	50.11	71,754	49.42	1,315,346	49.99	18,268	50.58	2,058,780
	Missing	–		–		–		–		–		–
Mother’s age, years												
	10 to 13	1802	0.14	18	0.12	252	0.17	4699	0.18	116	0.32	6887
	14 to 19	241,400	19.12	3221	20.68	26,988	18.59	528,369	20.08	8307	23.00	808,285
	20 to 34	893,614	70.79	11,017	70.74	103,308	71.15	1,873,715	71.21	24,230	67.09	2,905,884
	35 to 50	125,426	9.94	1318	8.46	14,632	10.08	224,201	8.52	3455	9.57	369,032
	Missing	44	0.00	1	0.01	11	0.01	121	0.00	6	0.02	183
Mother’s marital status												
	Married, stable union	17,126	1.36	225	1.44	2133	1.47	39,186	1.49	715	1.98	59,385
	Single	664,798	52.67	7792	50.03	93,570	64.45	1,462,749	55.59	16,737	46.34	2,245,646
	Divorced, widow	562,459	44.56	7444	47.79	48,238	33.22	1,111,178	42.23	18,576	51.44	1,747,895
	Missing	17,903	1.42	114	0.73	1250	0.86	17,992	0.68	86	0.24	37,345
Mother’s years of education												
	3 years or less	110,423	8.75	1522	9.77	19,982	13.76	348,072	13.23	9940	27.52	489,939
	4–7 years	448,705	35.55	5340	34.29	57,263	39.44	972,128	36.95	13,529	37.46	1,496,965
	8 years or more	683,650	54.16	8376	53.78	64,908	44.71	1,259,488	47.87	11,647	32.25	2,028,069
	Missing	19,508	1.55	337	2.16	3038	2.09	51,417	1.95	998	2.76	75,298
Mode of delivery												
	Cesarean	588,231	46.60	6377	40.94	52,874	36.42	971,728	36.93	6311	17.48	1,625,521
	Vaginal	672,947	53.31	9179	58.93	92,142	63.46	1,656,173	62.95	29,771	82.44	2,460,212
	Missing	1108	0.09	19	0.12	175	0.12	3204	0.12	32	0.09	4538
Prenatal consultations												
	None	11,400	0.90	241	1.55	2806	1.93	41,535	1.58	1642	4.55	57,624
	1 to 3	60,936	4.83	1162	7.46	13,145	9.05	227,280	8.64	7829	21.68	310,352
	4 to 6	345,996	27.41	5424	34.83	52,332	36.04	1,008,509	38.33	14,884	41.21	1,427,145
	7 or more	836,587	66.28	8646	55.51	75,561	52.04	1,335,183	50.75	11,281	31.24	2,267,258
	Missing	7367	0.58	102	0.65	1347	0.93	18,598	0.71	478	1.32	27,892
Household overcrowding												
	≤2 inhabitants per room	1,132,136	89.69	13,762	88.36	123,705	85.20	2,232,229	84.84	20,584	57.00	3,522,416
	> 2 inhabitants per room	61,694	4.89	817	5.25	11,687	8.05	197,927	7.52	10,849	30.04	282,974
	Missing	68,456	5.42	996	6.39	9799	6.75	200,949	7.64	4681	12.96	284,881
Rural / Urban area of residence												
	Rural	289,259	22.92	4436	28.48	33,382	22.99	826,070	31.40	26,302	72.83	1,179,449
	Urban	973,002	77.08	11,139	71.52	111,794	77.00	1,804,973	68.60	9812	27.17	2,910,720
	Missing	25	0.00	0	0.00	15	0.01	62	0.00	0	0.00	102

Overall, the prevalence rates of stunting and underweight was higher among children of Indigenous mothers (26.74 and 5.90%), followed by those born to Pardo (11.82 and 3.77%), Asian descent (10.99 and 3.64%), Black (10.41 and 3.48%), and White mothers (8.61 and 2.48%). The prevalence distribution for wasting and thinness was higher among children of Pardo mothers (5.36 and 5.52%), Asian descent (5.28 and 5.46%), Black (5.08 and 3.91%), Indigenous (4.19 and 4.20%), when compared to those of White mothers (3.70 and 3.91%) (Table Supl. 1). The descriptive statistics for age, anthropometric information, and the number of measurements is available in Supplementary Table 2.

**Fig. 2:**
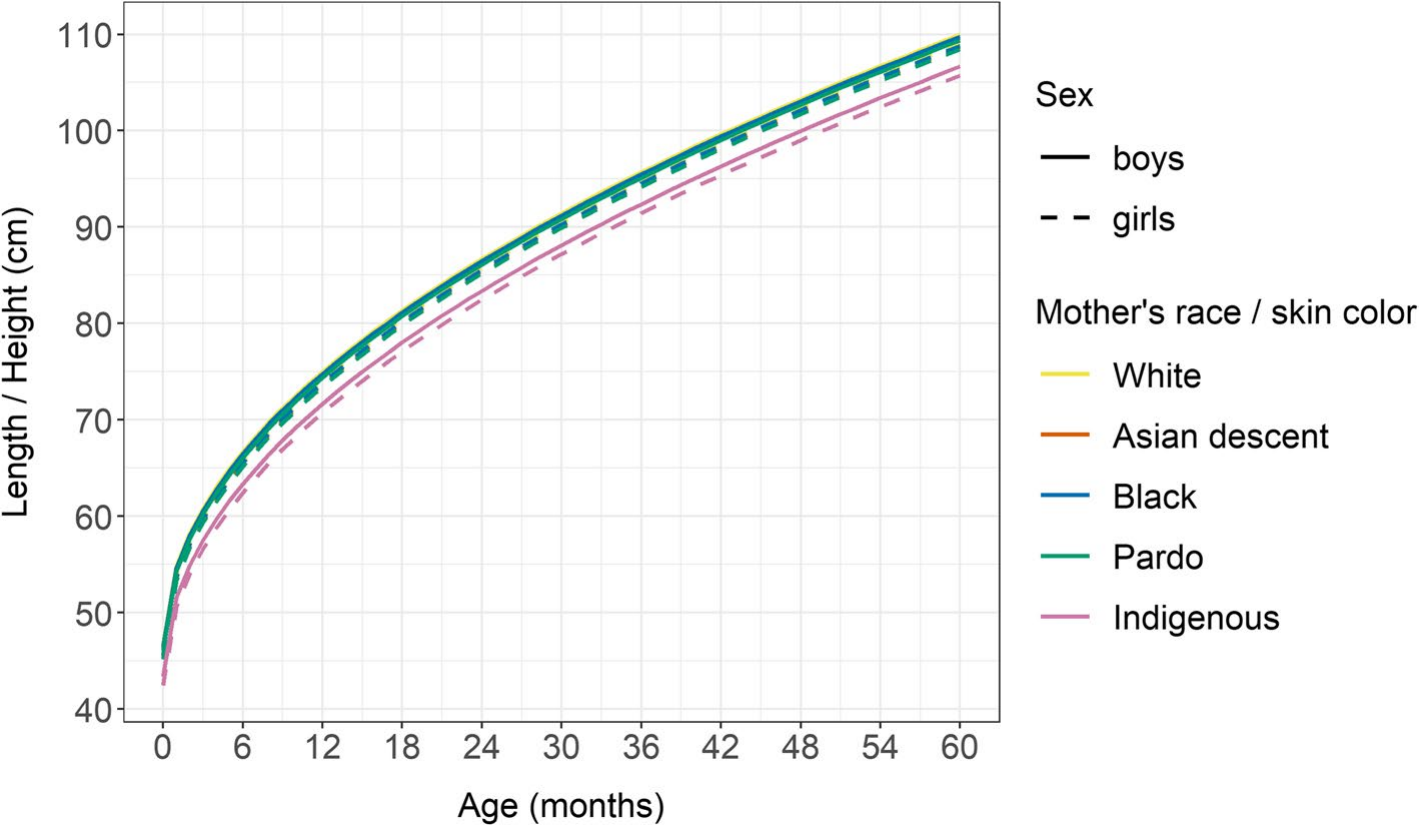
Estimated mean height according to sex and mother’s race / skin color. Brazil, 2008–2017

**Fig. 3:**
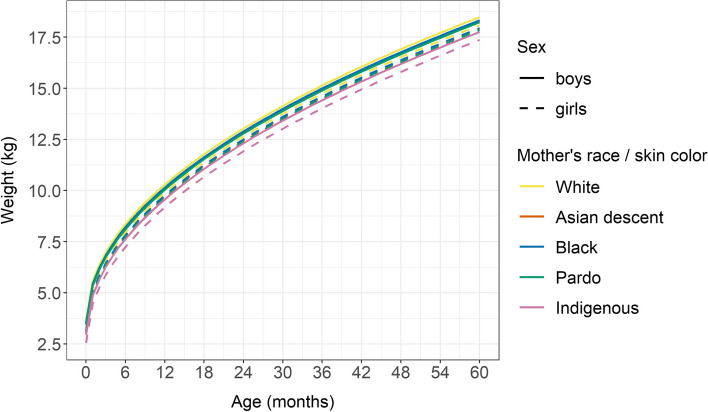
Estimated mean weight according to sex and mother’s race / skin color. Brazil, 2008–2017

Figures 2 and 3 display the estimated growth trajectories of both height/length (cm) and weight (kg) for age by sex. Fitted models do not include interactions of the available baseline factors with age as there were no indications of a significant result as such. The growth curve indicates that the mean weight and length/height of children born to mothers of each ethnoracial group studied exhibited comparatively less growth than their White counterparts, with more pronounced reductions evidenced in children born to Indigenous women (Figs. 2, 3).

The results obtained from our adjusted growth curve models indicate that children born to Indigenous mothers were on average 3.3 cm (95% CI: −3.36, −3.27) shorter than their White counterparts. Similarly, children born to Pardo (−0.60; 95% CI: −0.61, −0.59), Black (−0.21; 95% CI: −0.24, − 0.19) and Asian descent (− 0.39; 95% CI: − 0.46, − 0.32) mothers were shorter on average than those in the White group. In addition, compared to children of White women, those of Indigenous (− 0.74; 95% CI: − 0.76, − 0.72), Pardo (− 0.25; 95% CI: − 0.26, − 0.25), Black (− 0.15; 95% CI: − 0.16, − 0.14) and Asian descent (− 0.22; 95%CI: - 0.24, − 0.19) weighed less grams on average (Table Supl. 3).

Regarding WAZ and L/HAZ growth trajectories, a sharp decline in average z-scores was evidenced in the first weeks of life, followed by a period of recovery. The z-scores for most of the subgroups analyzed trended below zero across all ages. Growth patterns were similar in the groups, with children born to White women presenting the highest z-scores for WAZ and L/HAZ compared to children of Indigenous (WAZ -0.49; 95%CI: − 0.51, − 0.49; L/HAZ -0.87; 95%CI: − 0.88, − 0.85), Pardo (WAZ -0.17; 95%CI: − 0.17, − 0.17; L/HAZ -0.16; 95%CI: − 0.16, − 0.16) and Black (WAZ -0.11; 95%CI: − 0.11, − 0.10; L/HAZ -0.06; 95%CI: − 0.07, − 0.06) mothers, as well as those of Asian descent (WAZ -0.15; 95%CI: − 0.16, − 0.13; L/HAZ -0.11; 95%CI: − 0.12, − 0.09) (Tables 2, 3).

**Table 2:** Point and interval estimates for the parameters of the weight-for-age (WAZ) model. Brazil, 2008–2017

Parameter	Estimate	Standard Error	CI 95%
Intercept	−0.1588	0.0091	[−0.1767, − 0.1409]
Splines 1	− 0.3273	0.0161	[− 0.3590, − 0.2957]
Splines 2	− 0.2180	0.0086	[− 0.2348, − 0.2011]
Splines 3	0.0457	0.0097	[0.0267, 0.0647]
Splines 4	0.4605	0.0089	[0.4431, 0.4778]
Splines 5	0.4989	0.0090	[0.4813, 0.5165]
Splines 6	0.4744	0.0088	[0.4572, 0.4917]
Splines 7	0.3676	0.0088	[0.3503, 0.3848]
Splines 8	0.3038	0.0088	[0.2865, 0.3211]
Splines 9	0.2696	0.0089	[0.2522, 0.2869]
Splines 10	0.2464	0.0088	[0.2291, 0.2637]
Splines 11	0.2939	0.0121	[0.2701, 0.3177]
Sex (girls)	− 0.0329	0.0010	[−0.0349, − 0.0308]
Race / skin color (Asian descent)	−0.1474	0.0084	[− 0.1638, − 0.1309]
Race / skin color (Black)	− 0.1085	0.0029	[− 0.1142, − 0.1028]
Race / skin color (Pardo)	− 0.1727	0.0011	[− 0.1749, − 0.1705]
Race / skin color (Indigenous)	− 0.4986	0.0056	[− 0.5096, − 0.4876]
Educational level (3 years or less)	− 0.3217	0.0017	[− 0.3250, − 0.3184]
Educational level (4 to 7 years)	− 0.1603	0.0011	[− 0.1625, − 0.1581]
Civil status (single)	− 0.0253	0.0011	[− 0.0274, − 0.0233]
Civil status (divorced / widow)	0.0838	0.0054	[0.0732, 0.0945]
Mother’s age at birth	0.0060	0.0001	[0.0058, 0.0062]
σ_Intercept_	0.9576		
σ_ε_	0.6675		

Reference category: sex: boys; race/skin color: white; educational level: 8 years or more; marital status: married or in a stable union

The dimension of the splines accounts for the number of knots (K = 8) and the polynomial degree (*p* = 3)

**Table 3:** Point and interval estimates for the parameters of the length/height-for-age (L/HAZ) model. Brazil, 2008–2017

Parameter	Estimate	Standard Error	CI 95%
Intercept	− 0.6357	0.0144	[− 0.6639, − 0.6075]
Splines 1	− 0.5133	0.0260	[−0.5643, − 0.4624]
Splines 2	− 0.1505	0.0138	[− 0.1776, − 0.1234]
Splines 3	0.1967	0.0156	[0.1662, 0.2273]
Splines 4	0.3102	0.0143	[0.2822, 0.3382]
Splines 5	0.2356	0.0144	[0.2073, 0.2639]
Splines 6	0.0192	0.0141	[−0.0085, 0.0469]
Splines 7	0.3875	0.0142	[0.3598, 0.4153]
Splines 8	0.3635	0.0142	[0.3356, 0.3913]
Splines 9	0.4845	0.0142	[0.4566, 0.5125]
Splines 10	0.3844	0.0142	[0.3566, 0.4122]
Splines 11	0.4803	0.0194	[0.4422, 0.5184]
Sex (girls)	0.0399	0.0012	[0.0376, 0.0422]
Race / skin color (Asian descent)	−0.1063	0.0095	[−0.1250, − 0.0876]
Race / skin color (Black)	− 0.0651	0.0033	[− 0.0716, − 0.0587]
Race / skin color (Pardo)	− 0.1589	0.0013	[− 0.1614, − 0.1564]
Race / skin color (Indigenous)	− 0.8671	0.0064	[− 0.8796, − 0.8546]
Educational level (3 years or less)	− 0.3590	0.0019	[− 0.3628, − 0.3553]
Educational level (4 to 7 years)	− 0.1832	0.0013	[−0.1857, − 0.1807]
Civil status (single)	− 0.0423	0.0012	[− 0.0447, − 0.0400]
Civil status (divorced / widow)	0.0690	0.0062	[0.0569, 0.0810]
Mother’s age	0.0066	0.0001	[0.0064, 0.0068]
σ_Intercept_	0.9578		
σ_ε_	0.6675		

Reference category: sex: boys; race/skin color: white; educational level: 8 years or more; marital status: married or in a stable union

The dimension of the splines accounts for the number of knots (K = 8) and the polynomial degree (*p* = 3)

**Fig. 4:**
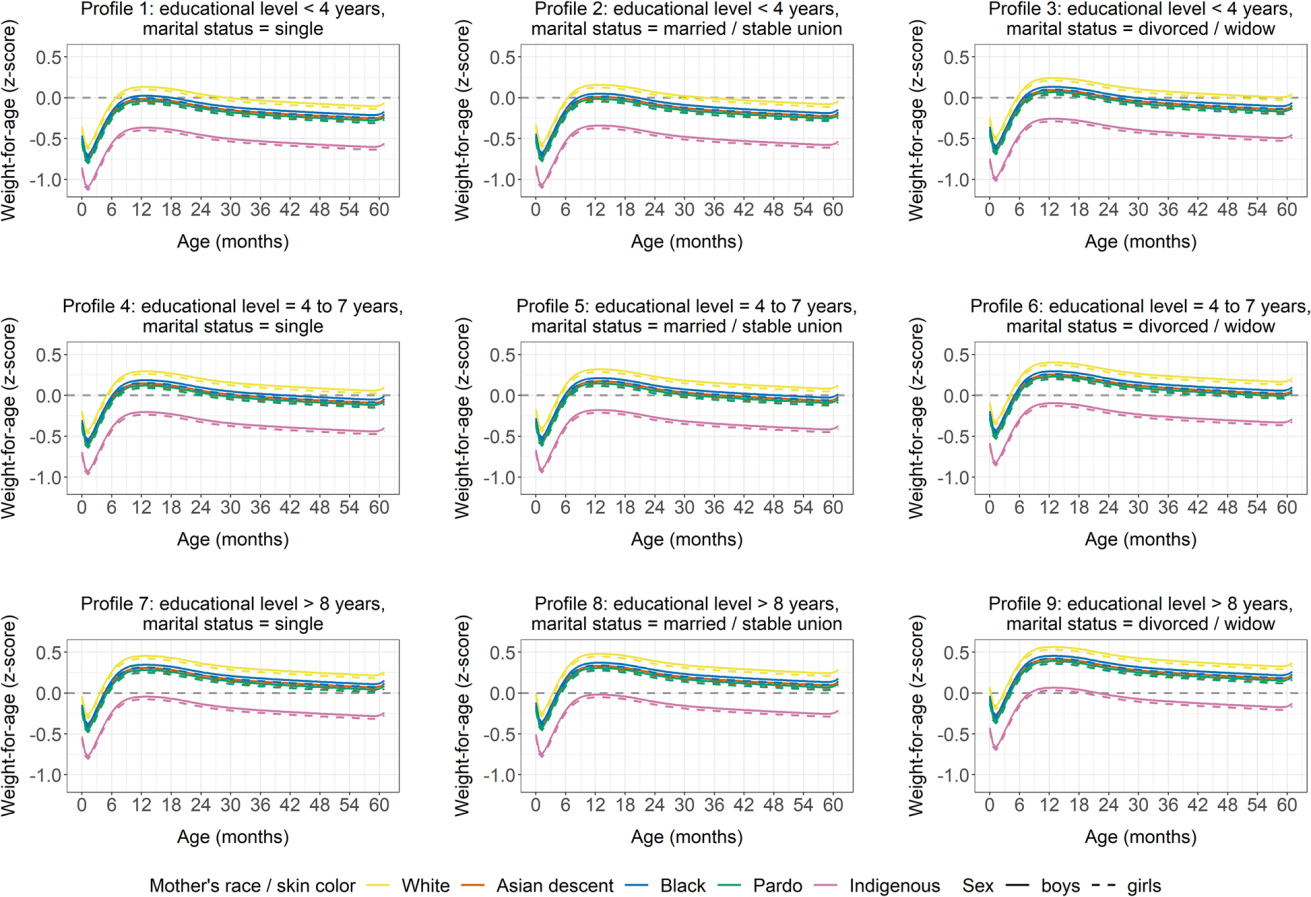
Estimated mean curves for weight-for-age z-scores model, according to mother’s age, educational level, and marital status. Brazil, 2008–2017

**Fig. 5:**
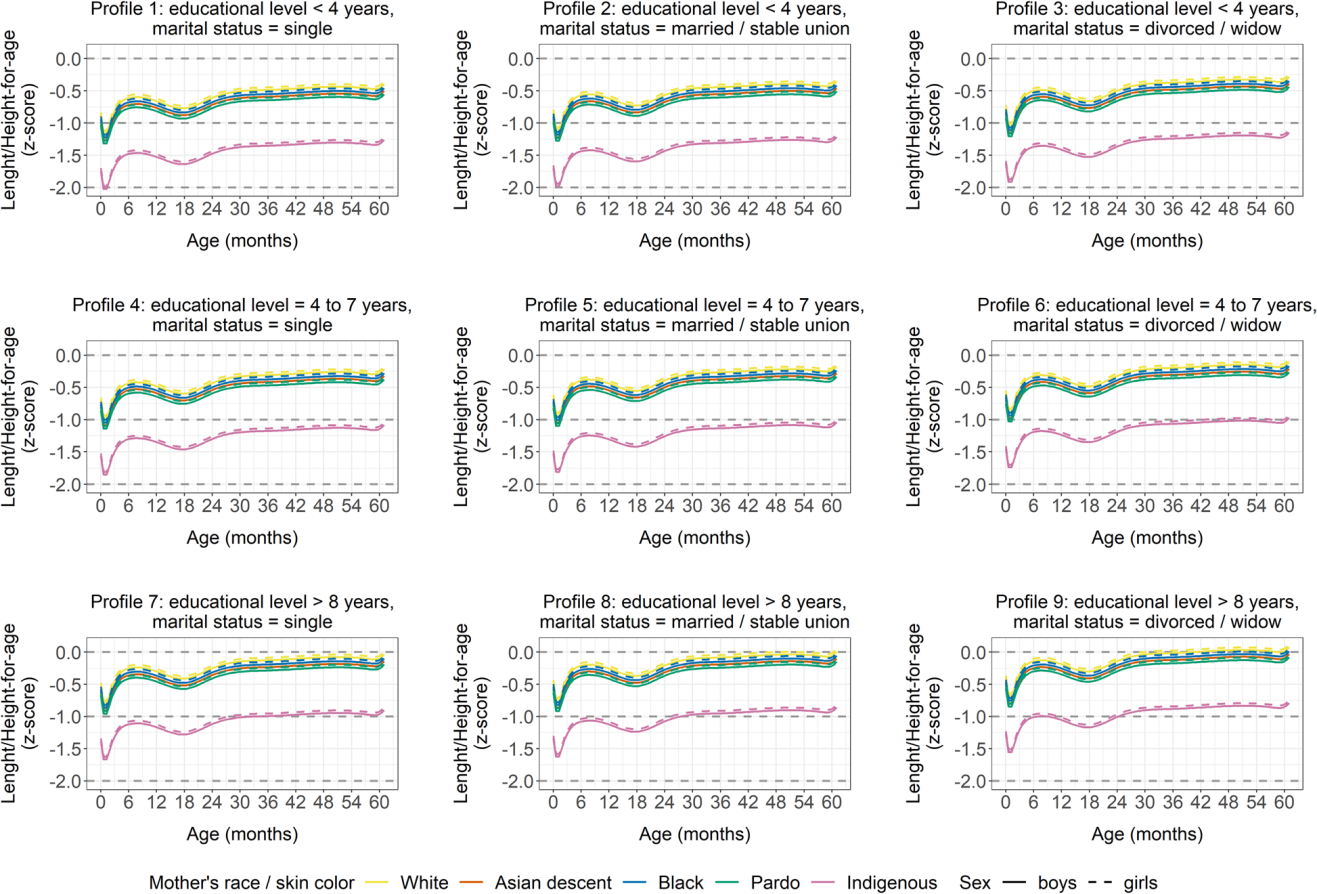
Estimated mean curves for length/height-for-age z-scores, according to mother’s age, educational level, and marital status. Brazil, 2008–2017

Our analysis indicated that, in general, growth trajectory outcomes were within the limits of normality per the WHO reference standard (± 2SD). However, when evaluating child growth trajectories in accordance with the sociodemographic characteristics of their mothers, children born to mothers facing greater social vulnerability (i.e., single mothers, with lower levels of education) presented less favorable results (Figs. 4 and 5).

Regarding the goodness of fit of the models, we verified based on the train and test analysis the models are well adjusted (Table Supl. 4, 5, 6, 7; Figure Supl. 1, 2, 3, 4).

## Discussion

In this study involving 4,090,271 individuals, we observed that those born to Indigenous mothers, as well as those born to Pardo, Black, and Asian descent women (to a lesser extent), exhibited less favorable growth outcomes compared to their White counterparts. High prevalence of stunting, underweight, thinness, and wasting were found across all ethnoracial groups, with the lower rates in children of White mothers. Disparities in mean weight and length/height for age persist between children of Indigenous women compared to White, although the z-score standardized growth trajectory remained within the limits of normality of the WHO reference standard for a general case (± 2SD). Our analysis indicated that social vulnerability further exacerbated the unfavorable growth trends observed in children born to mothers of ethnoracial background other than White.

The present study was conducted among the poorest population of a middle-income country with a history of major social and health inequalities, which may limit the generalizability of these findings. In this way, the ethnoracial distribution of our cohort may not be comparable to the Brazilian 2022 Census population [22], we found an underrepresentation of individuals who self-identified as White (30.86% vs 43.46%), Black (3.50% vs 10.17%), and Asian descent (0.38% vs 0.42%). Additionally, there was an overrepresentation of Pardo (64.33% vs 45.35%) and Indigenous (0.88% vs 0.60%) people [22].

In Brazil it has been observed a general decline in the prevalence of wasting and stunting among children under 5 years of age [7, 29, 30]. However, in our studied population, there is still a high burden of underweight, stunting, wasting, and thinness in children of Indigenous women, which aligns with the results of the First National Survey of Indigenous People’s Health and Nutrition in Brazil [31]. When stratified by ethnoracial groups a similar pattern was also observed in other Latin America countries, where Indigenous, Black, and Pardo children under 5 years old showed higher risk of stunting and wasting compared to White children [32]. Although nutritional studies on the population of Asian descent in Brazil are scarce, it is noteworthy the high prevalence of stunting, underweight, wasting, and thinness in this group in our study.

While previous studies conducted in Brazil have demonstrated persistent disparities in physical growth indicators by ethnoracial group, childhood growth assessments were limited by the lack of longitudinal anthropometric data [33–35]. When repeated measurements over time are available, we can provide more consistent estimates regarding specific periods of child growth, enabling the detection of variations and a better understanding of the growth trajectory [27]. Longitudinal growth in children is considered to be a reliable indicator of the quality of the environment in which they live and has been employed as a global indicator of quality of life [36].

Our growth models reinforce that even among the most socially disadvantaged population, racial disparities persist. And, similar to other low- and middle-income countries, Brazil has insufficiencies in providing appropriate nutrition and living conditions for the growth of children, with markedly racial inequalities, with unfavorable results concentrated among children born from Indigenous, followed by Black and Pardo mothers [31, 37].

Racism and its manifestations can explain the disadvantageous effects of ethnoracial inequalities on the physical growth of children through different pathways [23, 38]. Racism is a structural social determinant of health that modulates the living context and the health-disease process, establishing a continuum that, since colonial times in Brazil, has disproportionately impacted Black, Pardo and Indigenous populations [39].

Undoubtedly, a population’s health and nutrition conditions are inextricably linked to its respective social, economic, and environmental context [40], notably affected by racism [7, 41]. In line with this fact, our study revealed that maternal social vulnerability restrains child growth, as showed by the WAZ and L/HAZ open ward and downward shift in the growth trajectory curves when adjusted for mother’s age, educational level, and marital status. This draws even more attention to the children of Indigenous mothers, as, in general, this group remained below − 1 SD z-score for L/HAZ in vulnerability profiles.

In this regard, Indigenous populations confront significant disadvantages in maintaining sustainable food sources as the introduction and propagation of predatory natural resource management practices (e.g., lumber harvesting, deforestation, mining, etc.) strike at the very heart of their food systems. Exacerbating this situation, rampant malaria, mercury contamination [42], and infectious and parasitic diseases further limit the biological uptake of nutrients, placing Indigenous people at risk of developing a range of malnutrition manifestations, in particular, nutrient and micronutrient deficiencies, with a mortality hazard ratio for malnutrition reaching 16.39 (95%CI 12.88–20.85) when compared to children of White mothers [23, 39, 43–45].

It is known that the conditions one lives in determine the way of birth, growing up, illness, and dying. In this sense, children born to Indigenous, Black, and Pardo mothers will accumulate inequalities and vulnerabilities prior to birth, as well as the negative experiences caused by racism suffered during pregnancy [46, 47].

This scenario places Brazil on a difficult path to achieve the Sustainable Development Goals laid out by the United Nations before the 2030 target date.

### Study strengths and limitations

Our results provide valuable insight into early childhood growth trajectories among traditionally understudied racial/ethnic groups in Brazil; nonetheless, the present study has some limitations. Our study included records detailing complete length/height and weight information with biologically plausible values to improve accuracy. Notably, most of the individuals registered in the SISVAN database (≈68%) are beneficiaries of government assistance programs, which indicates an over-representation of poorer populations from smaller or rural municipalities and an under-representation of middle- and upper-class individuals residing in urban areas. Accordingly, the interpretation or generalization of the results presented herein warrants caution. Furthermore, the measurement of racism using the variable self-reported race/skin color constitutes a complex task, and data may vary according to whether an individual can self-classify or be effectively classified [48].

Nonetheless, our results present substantial evidence of the effects of ethnoracial disparities on children’s growth. To the best of our knowledge, this study represents the first use of a population-based database consisting of administrative data to study growth outcomes, incorporating an extensive range of anthropometric data collected over a five-year postnatal period.

## Conclusion

Our results reinforce that children born to vulnerable mothers, particularly Indigenous ones, experience systematically unfavorable physical growth compared to White children. Although prevalences for stunting, underweight, wasting, and thinness were high across all groups, a notable disparity exists in their distribution. These nutritional states reflect the unfavorable living conditions faced by these children. Recognizing racism as a central determinant of inadequate growth among some ethnoracial groups is an urgent priority to provide enhanced opportunities to thrive for minorities and historically neglected populations in Brazil. It is, therefore, of utmost importance to strengthen policies to protect Indigenous children to reduce the unacceptable large ethnoracial health inequalities observed. Future studies could investigate the inclusion of other social factors and geographical characteristics, which allow a better understanding of patterns of ethnoracial inequalities in child growth.

## Supplementary Information

**Additional file 1.**

## Data Availability

All data supporting this study were obtained from the Center for Data and Knowledge Integration for Health (CIDACS). These were licensed for exclusive use in the present study and, due to the privacy rules of the Brazilian Ethics Committee, are not openly available. Upon request with adequate justification and approval of an ethics committee, controlled access to data is evaluated; if possible, allowed access. Information on how to apply to access the data can be found at <https://cidacs.bahia.fiocruz.br/en/>. Requests to access the data should be directed to Helena B. M. da Silva at cidacs.curadoria@fiocruz.br.

## Abbreviations

BAZ: BMI-for-age z score
SUS: Brazilian Unified Health System
L/HAZ: Length/height for age z-score
SISVAN: Brazilian Food and Nutrition Surveillance System
WAZ: Weight-for-age z-score
WHZ: Weight-for-length/height z-score
