# Supplementary material for: Ethnoracial disparities in childhood growth trajectories in Brazil: a longitudinal nationwide study of four million children
Source: BMC Pediatr. 2024 Feb 10;24:103. doi: 10.1186/s12887-024-04550-3 (PMC10858530; doi:10.1186/s12887-024-04550-3)
Supplement: Supplementary file 1 — Additional file 1. [file 12887_2024_4550_MOESM1_ESM.docx]

**Supplementary material**

**Table Supl. 1:** Prevalence of child growth indicators according to mother’s race/skin color. Brazil, 2008-2017.

| **BAZ** | | | | | | | | | | |
| --- | --- | --- | --- | --- | --- | --- | --- | --- | --- | --- |
|  | Children of  White  mothers | % | Children of  Asian descent  mothers | % | Children of  Black  mothers | % | Children of  Pardo  mothers | % | Children of  Indigenous  mothers | % |
| Severe thinness | 92491 | 1.40 | 1507 | 2.06 | 12748 | 1.96 | 249299 | 2.11 | 2643 | 1.77 |
| Thinness | 165194 | 2.51 | 2485 | 3.40 | 21198 | 3.27 | 402055 | 3.41 | 3629 | 2.43 |
| Adequate | 4090329 | 62.13 | 45955 | 62.95 | 412674 | 63.60 | 7393831 | 62.65 | 90292 | 60.41 |
| Overweight risk | 1362149 | 20.69 | 13729 | 18.81 | 121070 | 18.66 | 2215449 | 18.77 | 34646 | 23.18 |
| Overweight | 593726 | 9.02 | 6122 | 8.39 | 53967 | 8.32 | 1018786 | 8.63 | 12993 | 8.69 |
| Obesity | 279274 | 4.24 | 3205 | 4.39 | 27152 | 4.18 | 523229 | 4.43 | 5271 | 3.53 |
| Total | 6583163 | 100.00 | 73003 | 100.00 | 648809 | 100.00 | 11802649 | 100.00 | 149474 | 100.00 |
| **L/HAZ** | | | | | | | | | | |
|  | Children of  White  mothers | % | Children of  Asian descent  mothers | % | Children of  Black  mothers | % | Children of  Pardo  mothers | % | Children of  Indigenous  mothers | % |
| Severe stunting | 181822 | 2.76 | 2730 | 3.74 | 23127 | 3.56 | 478034 | 4.05 | 13465 | 9.01 |
| Moderate stunting | 385439 | 5.85 | 5292 | 7.25 | 44423 | 6.85 | 917229 | 7.77 | 26509 | 17.73 |
| Adequate | 6015902 | 91.38 | 64981 | 89.01 | 581259 | 89.59 | 10407386 | 88.18 | 109500 | 73.26 |
| Total | 6583163 | 100.00 | 73003 | 100.00 | 648809 | 100.00 | 11802649 | 100.00 | 149474 | 100.00 |
| **WAZ** | | | | | | | | | | |
|  | Children of  White  mothers | % | Children of  Asian descent  mothers | % | Children of  Black  mothers | % | Children of  Pardo  mothers | % | Children of  Indigenous  mothers | % |
| Severe underweight | 27331 | 0.42 | 457 | 0.63 | 4087 | 0.63 | 77204 | 0.65 | 1666 | 1.11 |
| Moderate underweight | 135609 | 2.06 | 2196 | 3.01 | 18518 | 2.85 | 368674 | 3.12 | 7157 | 4.79 |
| Adequate | 6003845 | 91.20 | 66190 | 90.67 | 590412 | 91.00 | 10732755 | 90.94 | 136901 | 91.59 |
| Overweight | 416378 | 6.32 | 4160 | 5.70 | 35792 | 5.52 | 624016 | 5.29 | 3750 | 2.51 |
| Total | 6583163 | 100.00 | 73003 | 100.00 | 648809 | 100.00 | 11802649 | 100.00 | 149474 | 100.00 |
| **WHZ** | | | | | | | | | | |
|  | Children of  White  mothers | % | Children of  Asian descent  mothers | % | Children of  Black  mothers | % | Children of  Pardo  mothers | % | Children of  Indigenous  mothers | % |
| Severe wasting | 74878 | 1.14 | 1242 | 1.70 | 10599 | 1.63 | 208047 | 1.76 | 2243 | 1.50 |
| Wasting | 168382 | 2.56 | 2612 | 3.58 | 22366 | 3.45 | 424949 | 3.60 | 4027 | 2.69 |
| Adequate | 4214860 | 64.02 | 47505 | 65.07 | 426077 | 65.67 | 7662950 | 64.93 | 97330 | 65.12 |
| Overweight risk | 1333447 | 20.26 | 13303 | 18.22 | 117723 | 18.14 | 2151204 | 18.23 | 31480 | 21.06 |
| Overweight | 548941 | 8.34 | 5613 | 7.69 | 49195 | 7.58 | 919203 | 7.79 | 10439 | 6.98 |
| Obesity | 242655 | 3.69 | 2728 | 3.74 | 22849 | 3.52 | 436296 | 3.70 | 3955 | 2.65 |
| Total | 6583163 | 100 | 73003 | 100 | 648809 | 100.00 | 11802649 | 100.00 | 149474 | 100.00 |

Notes: BAZ: body mass index-for-age z-score, L/HAZ: length/height-for-age z-score, WAZ: weight-for-age z-score, WHZ: weight-for-length/height z-score.

**Table Supl. 2:** Descriptive statistics for anthropometric variables. Brazil, 2008-2017.

|  | Children of  White  mothers | | Children of  Asian descent  mothers | | Children of  Black  Mothers | | Children of  Pardo  mothers | | Children of  Indigenous  mothers | |
| --- | --- | --- | --- | --- | --- | --- | --- | --- | --- | --- |
|  | Mean (SD) | Min-Max | Mean (SD) | Min-Max | Mean (SD) | Min - Max | Mean (SD) | Min - Max | Mean (SD) | Min - Max |
| Age (months) | 28.96  (18.83) | 0 - 60.03 | 29.40  (19.07) | 0 - 60.03 | 30.11  (19.43) | 0 - 60.03 | 29.79  (19.23) | 0 - 60.03 | 30.30  (19.24) | 0 - 59.80 |
| Length/height (cm) | 92.32  (13.05) | 45 - 120 | 92.94  (12.53) | 45 - 120 | 93.85  (12.40) | 45 - 120 | 93.29  (12.10) | 45 - 120 | 91.47  (10.73) | 46 - 119 |
| Weight (kg) | 12.44  (5.34) | 0.50 – 36.30 | 12.25  (5.30) | 0.71 – 34.00 | 12.37  (5.43) | 0.52 – 35.80 | 12.25  (5.29) | 0.50 – 36.00 | 11.75  (5.00) | 0.51 – 34.50 |
| L/HAZ | -0.21  (1.42) | -6.00 - +6.00 | -0.30  (1.51) | -6.00 - +6.00 | -0.29  (1.48) | -6.00 - +6.00 | -0.37  (1.52) | -6.00 - +6.00 | -1.12  (1.55) | -6.00 - +6.00 |
| WAZ | 0.22  (1.15) | -5.99 - +5.00 | 0.07  (1.19) | -5.98 - +5.00 | 0.08  (1.18) | -5.93 - +5.00 | 0.03  (1.18) | -6.00 - +5.00 | -0.34  (1.12) | -5.96 - +4.99 |
| Number of measurements | 6.22  (5.24) | 2 - 109 | 5.69  (4.28) | 2 - 61 | 5.47  (3.99) | 2 - 77 | 5.49  (3.93) | 2 - 119 | 5.14  (3.16) | 2 - 66 |

Notes: SD: standard deviation, L/HAZ: length/height-for-age z-score, WAZ: weight-for-age z-score.

**Table Supl. 3:** Point and interval estimates for the parameters of both the height and weight model. Brazil, 2008-2017.

| Height | | | |  |
| --- | --- | --- | --- | --- |
| Parameter | Estimate | Standard Error | CI 95% |  |
| Intercept | 46.0288 | 0.0120 | [46.0052, 46.0523] |  |
| Race / skin color (Asian descent) | -0.3934 | 0.0356 | [-0.4631, -0.3237] |  |
| Race / skin color (Black) | -0.2148 | 0.0123 | [-0.2389, -0.1907] |  |
| Race / skin color (Pardo) | -0.6032 | 0.0048 | [-0.6126, -0.5938] |  |
| Race / skin color (Indigenous) | -3.3115 | 0.0238 | [-3.3581, -3.2648] |  |
| $\sigma_{\mathrm{Intercept}}$ | 3.8214 |  | [3.8191, 3.8237] |  |
| $\sigma_{\varepsilon}$ | 3.8419 |  | [3.8406, 3.8432] |  |
| Weight | | | |  |
| Intercept | 3.4134 | 0.0041 | [3.4054, 3.4214] |  |
| Race / skin color (Asian descent) | -0.2183 | 0.0123 | [-0.2442, -0.1925] |  |
| Race / skin color (Black) | -0.1517 | 0.0046 | [-0.1606, -0.1428] |  |
| Race / skin color (Pardo) | -0.2523 | 0.0018 | [-0.2557, -0.2488] |  |
| Race / skin color (Indigenous) | -0.7384 | 0.0088 | [-0.7559, -0.7211] |  |
| $\sigma_{\mathrm{Intercept}}$ | 1.4673 |  | [1.4678, 1.4683] | |
| $\sigma_{\varepsilon}$ | 1.4139 |  | [1.4134, 1.4143] | |

Reference category: Sex: Boys; Race/skin color: White; Educational level: 8 years or more; Marital status: Married or in a stable union. Adjusted for sex, $\sqrt{\mathrm{Age}}$, Mother’s educational level mother, Mother’s civil status, Mother’s age.

**Table Supl. 4:** Point and interval estimates for the parameters of the length / height model in the training dataset. SISVAN. 2008–2017.

| Parameter | Estimate | Standard Error | CI 95% |
| --- | --- | --- | --- |
| Intercept | 46.0302 | 0.0144 | [46.0021, 46.0583] |
| $\sqrt{\mathrm{Age}}$ | 8.1701 | 0.0010 | [8.1681, 8.1721] |
| Sex (girls) | -0.9290 | 0.0052 | [-0.9392, -0.9188] |
| Race / skin color (Asian descent) | -0.3757 | 0.0425 | [-0.4591, -0.2924] |
| Race / skin color (Black) | -0.2156 | 0.0147 | [-0.2444, -0.1868] |
| Race / skin color (Pardo) | -0.6039 | 0.0057 | [-0.6151, -0.5927] |
| Race / skin color (Indigenous) | -3.3262 | 0.0284 | [-3.3819, -3.2705] |
| Educational level (3 years or less) | -1.3929 | 0.0085 | [-1.4096, -1.3761] |
| Educational level (4 to 7 years) | -0.6975 | 0.0056 | [-0.7085, -0.6864] |
| Civil status (single) | -0.1725 | 0.0054 | [-0.1830, -0.1620] |
| Civil status (divorced / widow) | 0.2356 | 0.0275 | [0.1817, 0.2894] |
| Mother’s age | 0.0260 | 0.0004 | [0.0252, 0.0269] |
| $\sigma_{\mathrm{Intercept}}$ | 3.8208 |  | [3.8170, 3.8246] |
| $\sigma_{\varepsilon}$ | 3.8425 |  | [3.8409, 3.8442] |

Reference category: Sex: Boys; Race/skin color: White; Educational level: 8 years or more; Marital status: Married or in a stable union. Adjusted for sex, $\sqrt{\mathrm{Age}}$, Mother’s educational level mother, Mother’s civil status, Mother’s age.

**Table Supl. 5:** Point and interval estimates for the parameters of the weight model in the training dataset. SISVAN. 2008–2017.

| Parameter | Estimate | Standard Error | CI 95% |
| --- | --- | --- | --- |
| Intercept | 3.4110 | 0.0049 | [3.4015, 3.4205] |
| $\sqrt{\mathrm{Age}}$ | 1.9110 | 0.0001 | [1.9107, 1.9113] |
| Sex (girls) | -0.3906 | 0.0019 | [-0.3944, -0.3868] |
| Race / skin color (Asian descent) | -0.2493 | 0.0158 | [-0.2802, -0.2184] |
| Race / skin color (Black) | -0.1508 | 0.0054 | [-0.1615, -0.1402] |
| Race / skin color (Pardo) | -0.2540 | 0.0021 | [-0.2581, -0.2498] |
| Race / skin color (Indigenous) | -0.7336 | 0.0105 | [-0.7542, -0.7130] |
| Educational level (3 years or less) | -0.4361 | 0.0032 | [-0.4423, -0.4299] |
| Educational level (4 to 7 years) | -0.2171 | 0.0021 | [-0.2212, -0.2130] |
| Civil status (single) | -0.0215 | 0.0020 | [-0.0254, -0.0176] |
| Civil status (divorced / widow) | 0.1375 | 0.0102 | [0.1176, 0.1575] |
| Mother’s age | 0.0098 | 0.0002 | [0.0095, 0.0101] |
| $\sigma_{\mathrm{Intercept}}$ | 1.4678 |  | [1.4671, 1.4684] |
| $\sigma_{\varepsilon}$ | 1.4142 |  | [1.4138, 1.4146] |

Reference category: Sex: Boys; Race/skin color: White; Educational level: 8 years or more; Marital status: Married or in a stable union. Adjusted for sex, $\sqrt{\mathrm{Age}}$, Mother’s educational level mother, Mother’s civil status, Mother’s age.

**Table Supl. 6:** Point and interval estimates for the parameters of the L/HAZ model in the training dataset. SISVAN. 2008–2017.

| Parameter | Estimate | Standard Error | CI 95% |
| --- | --- | --- | --- |
| Intercept | -0.6503 | 0.0172 | [-0.6840, -0.6166] |
| Splines 1 | -0.4998 | 0.0311 | [-0.5607, -0.4389] |
| Splines 2 | -0.1326 | 0.0165 | [-0.1650, -0.1002] |
| Splines 3 | 0.2128 | 0.0187 | [0.1762, 0.2493] |
| Splines 4 | 0.3204 | 0.0171 | [0.2869, 0.3539] |
| Splines 5 | 0.2543 | 0.0173 | [0.2205, 0.2882] |
| Splines 6 | 0.0333 | 0.0169 | [0.0002, 0.0665] |
| Splines 7 | 0.4031 | 0.0170 | [0.3699, 0.4364] |
| Splines 8 | 0.3778 | 0.0170 | [0.3445, 0.4111] |
| Splines 9 | 0.4993 | 0.0170 | [0.4659, 0.5326] |
| Splines 10 | 0.3983 | 0.0170 | [0.3651, 0.4316] |
| Splines 11 | 0.4984 | 0.0232 | [0.4529, 0.5440] |
| Sex (girls) | 0.0409 | 0.0014 | [0.0382, 0.0436] |
| Race / skin color (Asian descent) | -0.1003 | 0.0114 | [-0.1226, -0.0779] |
| Race / skin color (Black) | -0.0658 | 0.0039 | [-0.0735, -0.0581] |
| Race / skin color (Pardo) | -0.1591 | 0.0015 | [-0.1621, -0.1561] |
| Race / skin color (Indigenous) | -0.8711 | 0.0076 | [-0.8860, -0.8561] |
| Educational level (3 years or less) | -0.3583 | 0.0023 | [-0.3628, -0.3538] |
| Educational level (4 to 7 years) | -0.1832 | 0.0015 | [-0.1862, -0.1803] |
| Civil status (single) | -0.0418 | 0.0014 | [-0.0446, -0.0390] |
| Civil status (divorced / widow) | 0.0655 | 0.0074 | [0.0511, 0.0799] |
| Mother’s age | 0.0066 | 0.0001 | [0.0064, 0.0068] |
| $\sigma_{\mathrm{Intercept}}$ |  |  |  |
| $\sigma_{\varepsilon}$ |  |  |  |

Reference category: Sex: Boys; Race/skin color: White; Educational level: 8 years or more; Marital status: Married or in a stable union. Adjusted for sex, Age (splines), Mother’s educational level mother, Mother’s civil status, Mother’s age. Notes: The dimension of the splines accounts for the number of knots (K=8) and the polynomial degree (p = 3).

**Table Supl. 7:** Point and interval estimates for the parameters of the WAZ model in the training dataset. SISVAN. 2008–2017

| Parameter | Estimate | Standard Error | CI 95% |
| --- | --- | --- | --- |
| Intercept | -0.1618 | 0.0109 | [-0.1832, -0.1404] |
| Splines 1 | -0.3392 | 0.0193 | [-0.3770, -0.3015] |
| Splines 2 | -0.2102 | 0.0103 | [-0.2303, -0.1901] |
| Splines 3 | 0.0477 | 0.0116 | [0.0250, 0.0704] |
| Splines 4 | 0.4630 | 0.0106 | [0.4422, 0.4838] |
| Splines 5 | 0.5015 | 0.0107 | [0.4805, 0.5226] |
| Splines 6 | 0.4771 | 0.0105 | [0.4565, 0.4977] |
| Splines 7 | 0.3704 | 0.0105 | [0.3498, 0.3911] |
| Splines 8 | 0.3040 | 0.0106 | [0.2833, 0.3247] |
| Splines 9 | 0.2741 | 0.0106 | [0.2533, 0.2948] |
| Splines 10 | 0.2471 | 0.0105 | [0.2264, 0.2678] |
| Splines 11 | 0.3034 | 0.0145 | [0.2750, 0.3319] |
| Sex (girls) | -0.0321 | 0.0012 | [-0.0345, -0.0297] |
| Race / skin color (Asian descent) | -0.1493 | 0.0100 | [-0.1689, -0.1296] |
| Race / skin color (Black) | -0.1086 | 0.0035 | [-0.1154, -0.1018] |
| Race / skin color (Pardo) | -0.1729 | 0.0013 | [-0.1755, -0.1702] |
| Race / skin color (Indigenous) | -0.5000 | 0.0067 | [-0.5131, -0.4869] |
| Educational level (3 years or less) | -0.3214 | 0.0020 | [-0.3253, -0.3174] |
| Educational level (4 to 7 years) | -0.1597 | 0.0013 | [-0.1623, -0.1571] |
| Civil status (single) | -0.0248 | 0.0013 | [-0.0273, -0.0224] |
| Civil status (divorced / widow) | 0.0853 | 0.0065 | [0.0726, 0.0980] |
| Mother’s age | 0.0060 | 0.0001 | [0.0058, 0.0062] |
| $\sigma_{\mathrm{Intercept}}$ | 0.9577 |  | [0.9575, 0.9578] |
| $\sigma_{\varepsilon}$ | 0.6674 |  | [0.6672, 0.6677] |

Reference category: Sex: Boys; Race/skin color: White; Educational level: 8 years or more; Marital status: Married or in a stable union. Adjusted for sex, Age (splines), Mother’s educational level mother, Mother’s civil status, Mother’s age. Notes: The dimension of the splines accounts for the number of knots (K=8) and the polynomial degree (p = 3).

**
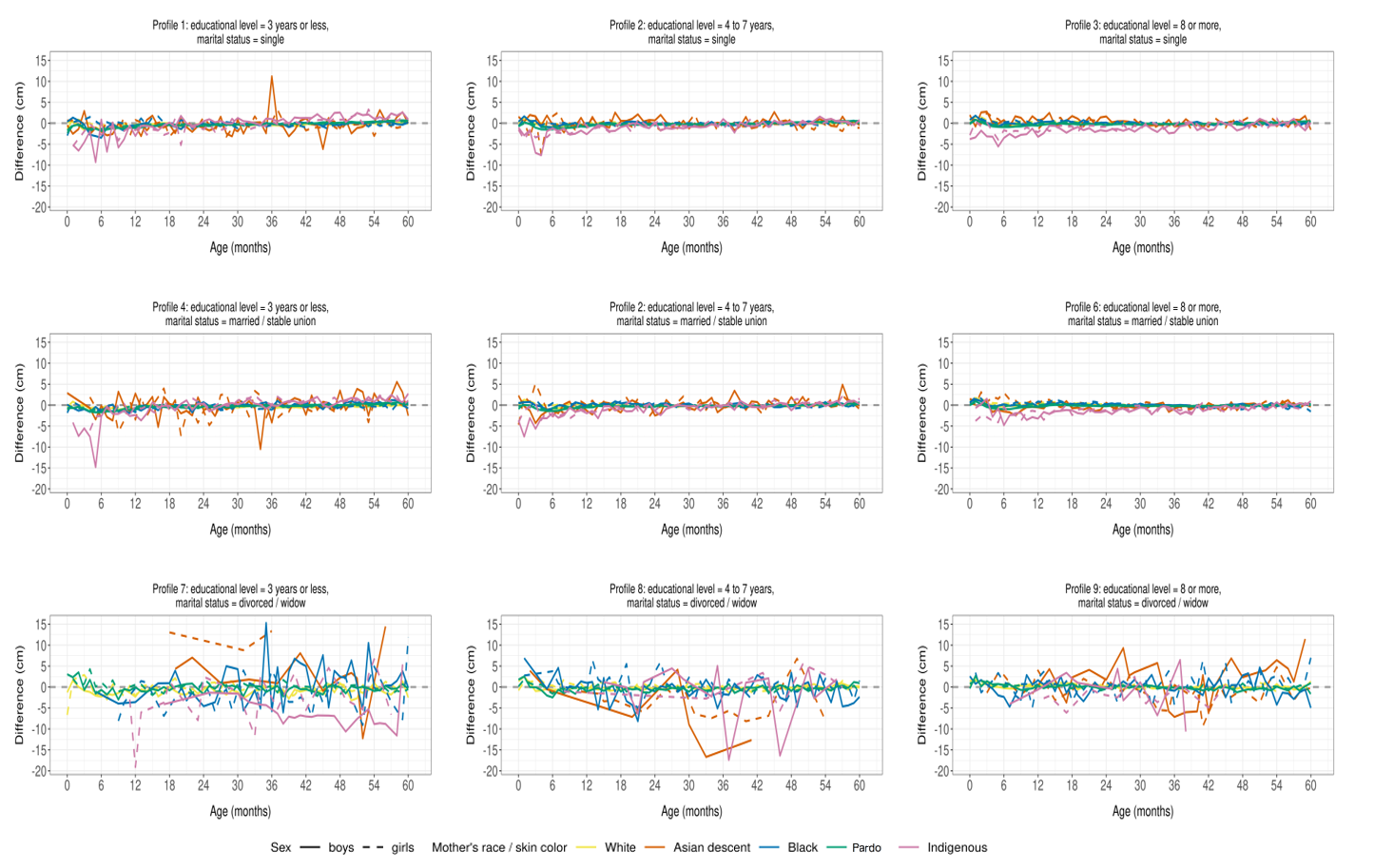
Figure S1:** Difference between estimated and observed mean height curves for the testing dataset, based on parameter estimates of the training dataset.


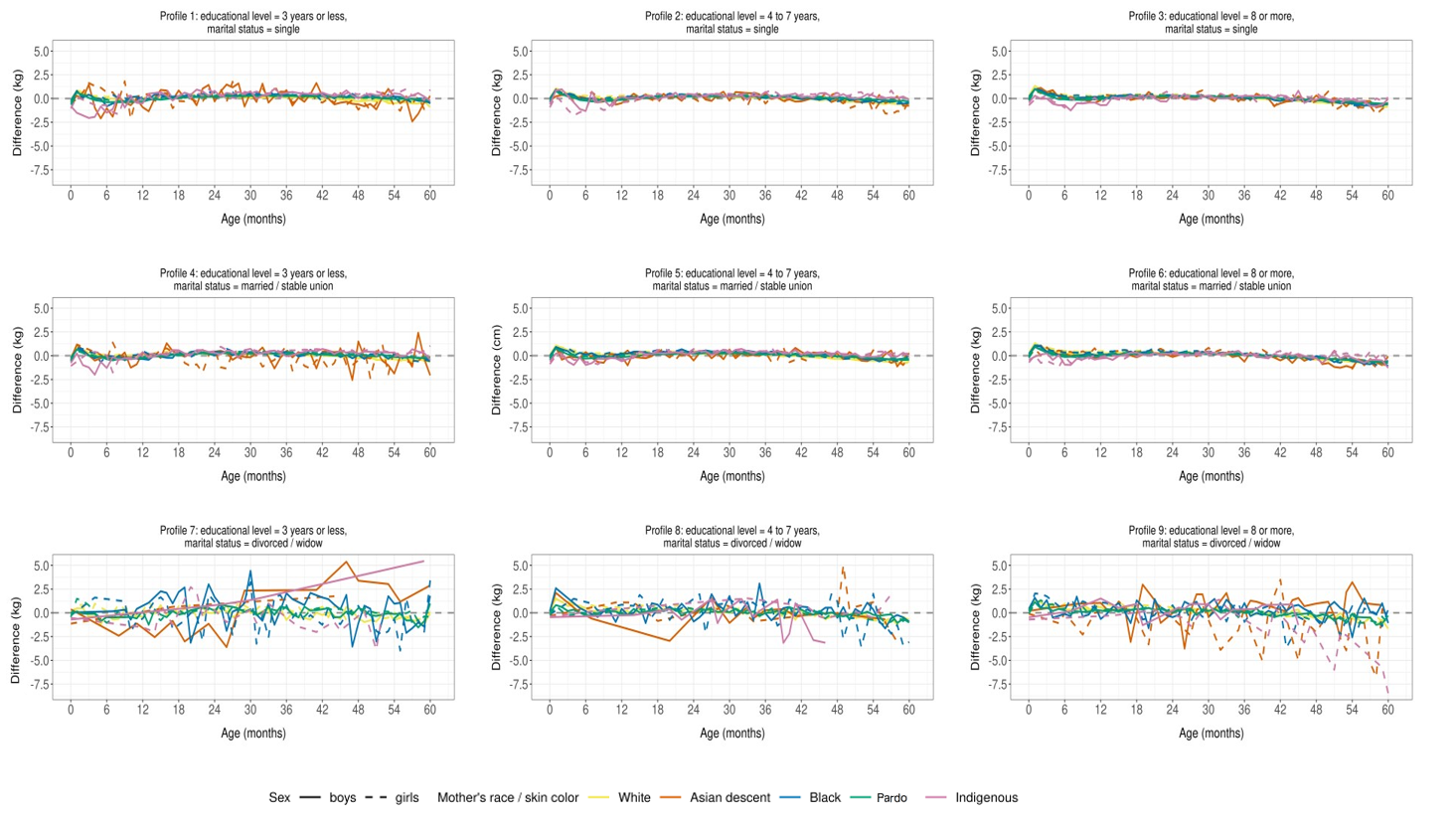
**Figure S2:** Difference between estimated and observed mean weight curves for the testing dataset, based on parameter estimates of the training dataset.

***
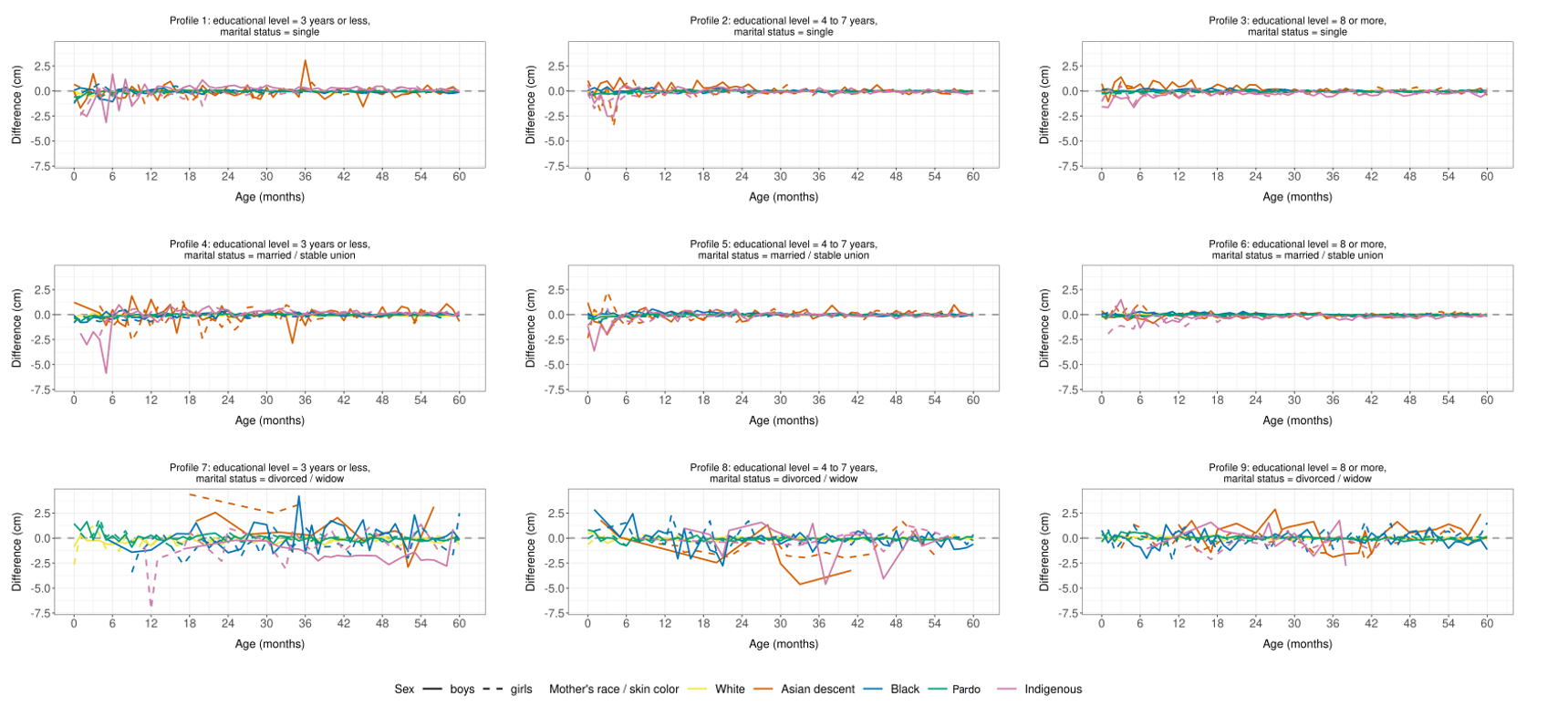
***

**Figure S3:** Difference between estimated and observed mean L/HAZ curves for the testing dataset, based on parameter estimates of the training dataset.


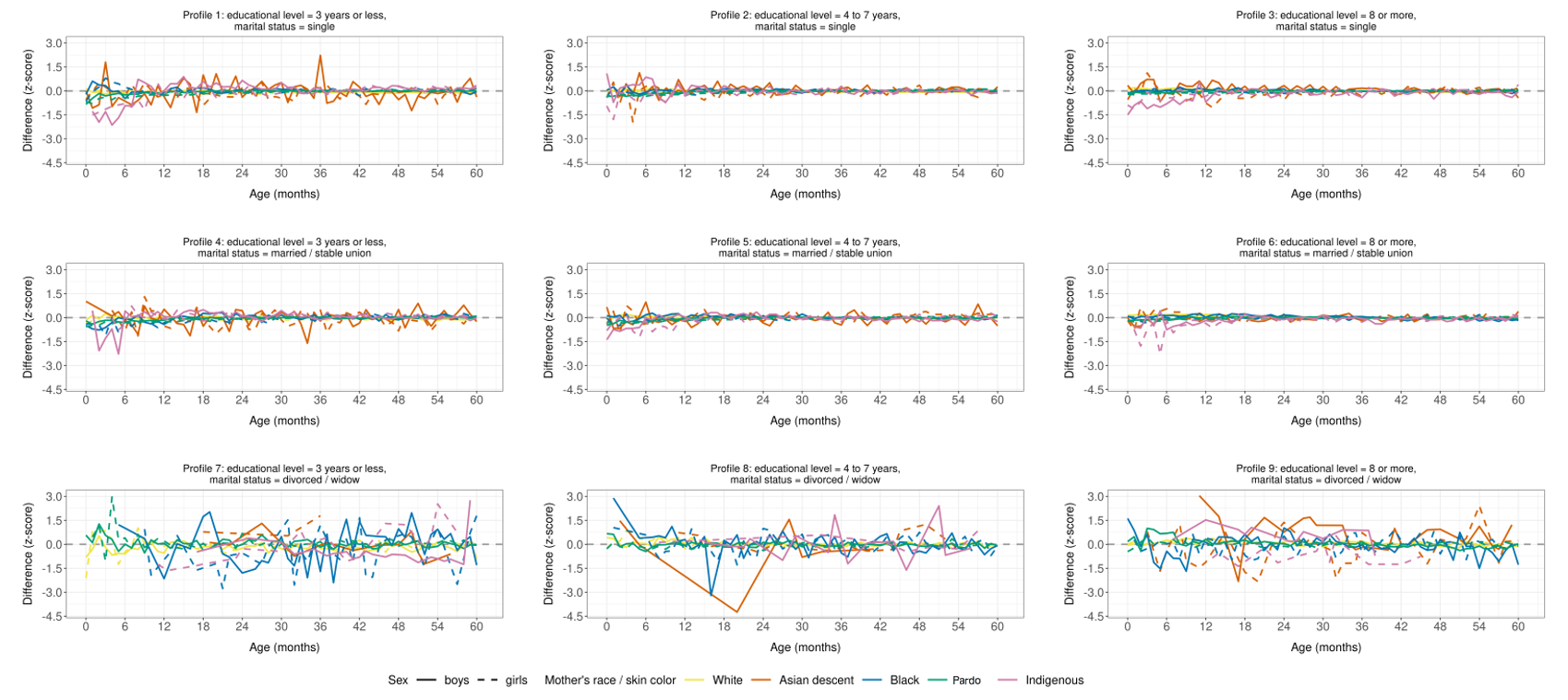


**Figure S4:** Difference between estimated and observed mean WAZ curves for the testing dataset, based on parameter estimates of the training dataset.
